# Insights on SNPs of Human Activation-Induced Cytidine Deaminase AID

**DOI:** 10.3390/ijms26136107

**Published:** 2025-06-25

**Authors:** Ekaterina A. Koveshnikova, Aleksandra A. Kuznetsova

**Affiliations:** Institute of Chemical Biology and Fundamental Medicine, Siberian Branch of Russian Academy of Sciences, 630090 Novosibirsk, Russia; ekat.koveshnikova@gmail.com

**Keywords:** human activation-induced cytidine deaminase AID, single-nucleotide polymorphism SNP, in silico evaluation of SNPs effect, effect of amino acid substitutions on enzyme functioning

## Abstract

DNA-deaminase AID plays a pivotal role in adaptive immunity, antibody diversification and epigenetic regulation. AID catalyzes cytidine deamination in immunoglobulin genes, facilitating somatic hypermutation (SHM), class-switch recombination (CSR) and gene conversion (GC). However, the dysregulation of AID activity can lead to oncogenic mutations and immune disorders such as hyper-IgM syndrome type 2 (HIGM2). At present the number of studies investigating the role of AID polymorphic variants in the promotion of pathology is low. The current review examines the structural and functional aspects of AID, focusing on the impact of amino acid substitutions—both natural polymorphisms and artificial mutations—on its catalytic activity, substrate binding and interactions with regulatory proteins. Additionally, a bioinformatic analysis of single-nucleotide polymorphisms of AID deposited in the dbSNP database was performed. SNPs leading to amino acid substitutions in the primary protein structure were analyzed. The bioinformatic analysis of SNPs in the AID gene predicts that among 208 SNPs causing amino acid substitutions in the primary protein structure, 62 substitutions may have significant negative impact on the functioning of AID. The integration of computational predictions with experimental data underscores the importance of AID regulation in maintaining immune homeostasis and highlights potential markers for immune-related pathologies. This comprehensive analysis provides insights into the molecular mechanisms of AID dysfunction and its implications for disease.

## 1. Introduction

**Figure 1:**
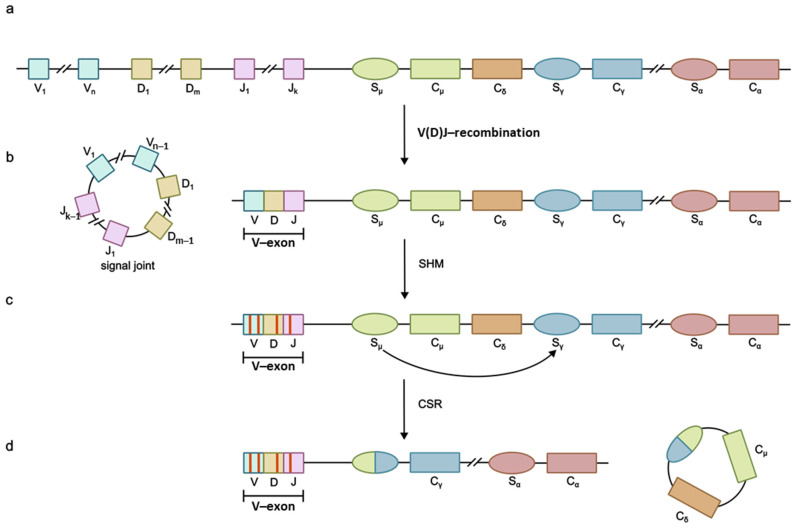
Schematic representation of immunoglobulin heavy chain (IgH) gene rearrangement. (**a**) Germline configuration of the IgH locus, showing the unrearranged segments encoding the constant (C), variable (V), joining (J) and diversity (D) domains. (**b**) V(D)J recombination. RAG enzymes mediate the excision of intervening DNA between V, D and J segments (forming circular signal joints) to generate a functional V-exon, which encodes the antigen-binding variable domain. (**c**) Somatic hypermutation. AID introduces point mutations (red lines) into the rearranged V-gene. Mutations are concentrated in complementarity-determining regions (CDRs). (**d**) Class-switch recombination. The region coding for the constant domain of one isotype of immunoglobulin is switched to the constant domain gene of another isotype (aswitch from IgM to IgG is shown). AID-induced double-strand breaks in switch (S) regions upstream of C genes (e.g., Sμ→Sγ) enable isotype switching (e.g., IgM to IgG) via NHEJ, altering effector functions while retaining antigen specificity.

The human cytidine deaminase APOBEC (apolipoprotein B mRNA editing enzyme, catalytic polypeptide-like) superfamily includes APOBEC1 (A1), APOBEC2 (A2), APOBEC3 (A3), APOBEC4 (A4) families, as well as activation-induced cytidine deaminase (AID). APOBEC enzymes play a crucial part in lipid metabolism, antibody diversification and host protection from viral infections. DNA-deaminase AID participates in the adaptive immune response by initiating antibody diversification processes such as somatic hypermutation (SHM), class-switch recombination (CSR) and gene conversion (GC) in some species. These processes, alongside V(D)J recombination catalyzed by RAG enzymes, generate an antibody repertoire whose size significantly exceeds the size genome [1]. During the V(D)J recombination proceeding during the early stages of lymphocyte differentiation, genes that code for variable (V) regions of immunoglobulins are rearranged (Figure 1), thereby increasing antibody diversity [2]. SHM, in its turn, occurs in dividing B-lymphocytes in the germinal centers of secondary lymphoid organs (the spleen and lymphatic nodes) during immune response and is necessary for antibody affinity maturation, i.e., the increase in antibody affinity for antigens. During SHM the number of mutations in V-regions of immunoglobulin genes is increased (Figure 1c), in particular in hypervariable complementarity determining regions (CDRs), playing a crucial role in antigen recognition and binding. CSR is the process of switching between the constant (C) domain genes of the heavy immunoglobulin chain (Figure 1d), which alters the antibody isotypes produced by the cell while preserving their specificity and affinity for the antigen [3]. Gene conversion occurs in the bursa of the Fabricius of birds, as well as in a number of other species [4] and involves V-region pseudogenes [5].

**Figure 2:**
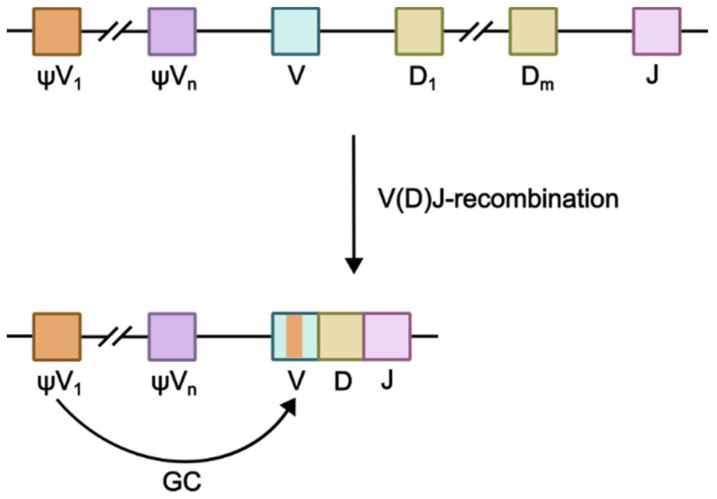
Gene conversion scheme. Following V(D)J recombination, AID introduces targeted DNA lesions into the rearranged V-exon, subsequently repaired through homologous recombination using upstream pseudogene templates (ψV_1_–ψVn), thereby diversifying the antibody repertoire while maintaining framework region integrity.

The molecular basis of SHM, CSR and GC involves AID-catalyzed cytidine residue deamination in immunoglobulin genes, producing 2′-deoxyuridine’s formation, recognized by repair systems as a lesion. Uracil in the U:G base pair may be removed from DNA via base excision repair (BER) or mismatch repair (MMR) pathways. During SHM, error-prone polymerases such as Rev1 and Polθ fill the resulting gaps [1]. In CSR, double-stranded breaks in switch (S) regions between immunoglobulin constant-domain genes are repaired via a joined non-homologous end joining (NHEJ) mechanism resulting in the deletion of the fragment between two S-regions [6]. During gene conversion, AID-induced lesions are repaired through homologous recombination using upstream pseudo-V-genes (ψV) as a template (Figure 2) [5].

Another important biological process involving DNA-deaminases AID/APOBEC is the active demethylation of the epigenetic mark 5-methylcytosine (5meC). It should be noted that DNA-methyltransferase enzymes DNMT3a and DNMT3b catalyzing epigenetic DNA methylation in mammals are well-characterized, while the enzymes responsible for demethylation remained unknown until the discovery of mononuclear non-heme Fe(II)-dependent dioxygenases of the TET (ten-eleven translocation) family [7,8]. TET enzymes oxidize the methyl group of 5meC, while AID/APOBEC enzymes deaminate these cytosine derivatives, forming methylated uracil derivatives, which, in their turn, are removed from DNA through the base excision repair pathway. The processes of the demethylation of the paternal genome in the fertilized egg cell, the imprint erasure in embryonic cells, as well as the initiation of tissue-specific gene expression, proceed via the active DNA demethylation pathway [9,10]. Thus, AID/APOBEC enzyme activity is essential for cellular function and viability.

However, the ability of APOBEC family enzymes to deaminate cytidine in the single-stranded DNA is a double-edged sword of their functioning. Dysregulation of their selectivity or cellular control may lead to increased mutation levels in genome DNA, promoting oncogenic transformation. Under normal physiological conditions, AID expression is transient and restricted to antigen-activated B-lymphocytes in germinal centers [11]. At the same time AID is constitutively expressed in human B-cell non-Hodgkin lymphomas, in particular in follicular lymphoma, diffuse large B-cell lymphoma and Burkitt lymphoma [12,13,14]; there has also been discovered a high level of AID expression in B-cells during chronic lymphocytic leukemia [15]. It was shown that AID is capable of inducing double-stranded genome DNA breaks, leading to chromosomal translocations between IgH genes and oncogenes: c-myc in Burkitt lymphoma [16,17,18], BCL2 and BCL6 in diffuse large B-cell lymphoma [19,20] and CCND1 in mantle cell lymphoma [18]. In addition, AID-induced hypermutations in oncogenes PIM1, MYC, RhoH/TTF (ARHH) and PAX5 are observed in more than 50% of diffuse large B-cell lymphomas [21]. Mutations in the *AICDA* gene leading to AID inactivation are associated with hyper-IgM syndrome type 2 (HIGM2), which is an immunodeficiency type characterized by increased IgM class antibody content in blood, accompanied by the almost complete absence of IgG, IgE and IgA class antibodies [22].

Given these dual roles, a strict regulation of AID/APOBEC activity is vital for adaptive immunity and epigenetic demethylation. The appearance of enzyme variants with altered activity may be a consequence of naturally occurring point mutations in the coding gene, a single-nucleotide polymorphism (SNP). SNPs in the AID gene are of critical importance due to their potential to alter enzymes’ function, with far-reaching implications for immune regulation, disease susceptibility and therapeutic development. Dysregulated AID activity due to SNPs can disrupt AID’s role in SHM and CSR, leading to immunodeficiency or defective antibody responses; as well, it may promote oncogenic mutations in MYC, BCL2 or PAX5, contributing to B-cell lymphomas. SNPs in catalytic, DNA-binding or regulatory domains can affect residues critical for AID’s function and interaction with its partners. SNPs linked to HIGM2 or lymphoma susceptibility could aid in early diagnosis and personalized treatment; moreover, combining computational predictions with experimental assays can clarify its pathogenicity. By elucidating how genetic variants perturb AID’s roles in immunity and genome integrity, we can improve genetic screening for immune disorders and cancers, develop targeted therapies to correct or compensate for pathogenic variants and uncover broader principles of enzyme regulation applicable to other APOBEC family members.

The NCBI dbSNP database (https://www.ncbi.nlm.nih.gov/snp/) (accessed on 18 September 2024) contains information about more than 4500 SNPs in the gene of AID. In this review, we focus on an analysis of natural polymorphic and artificial mutant variants of AID with known relationships between amino acid substitutions and efficient functioning, alongside a bioinformatic assessment of uncharacterized variants. Presently there exists a large number of bioinformatic approaches that allow one to predict the effect of single-nucleotide substitutions on protein properties. SNPs leading to amino acid substitutions in the AID primary structure have been analyzed using the dbNSFP 4.7a database [23,24] and a number of applications: SIFT4G [25], PolyPhen-2 HDIV и PolyPhen-2 HVAR [26], PROVEAN [27], MetaSVM/MetaLR [28], M-CAP [29], REVEL [30], CADD [31], DANN [32]. Based on multiple alignments of the different species of AID using the ConSurf Server [33] the functional and structural importance of amino acid substitutions caused by SNPs (i.e., amino acid residue conservation) was estimated. The Clustal Omega 2.1 algorithm was used for multiple sequence alignment [34]. Using different techniques for predicting the SNP effects increases the analysis accuracy and the evaluation of SNPs’ hypothetical capability to affect protein functions.

## 2. Aid Structure and Catalysis Mechanism

All AID/APOBEC family enzymes share a common structural organization with zinc-dependent deaminases. This family includes tRNA adenosine deaminases (Tad/ADAT2) that convert adenosine to inosine in the anticodon of different tRNAs in both eukaryotes and prokaryotes and that are considered to be the origin of AID/APOBEC family genes [35,36].

**Figure 3:**
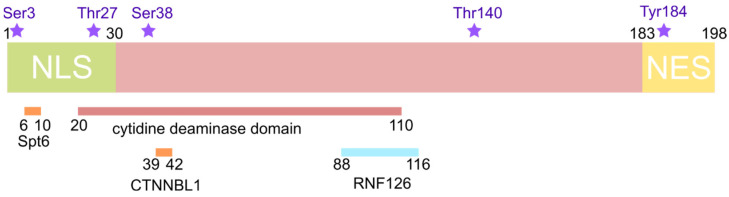
Structural organization of AID. Phosphorylation sites are highlighted in purple.

A number of regions playing a certain role in the functioning of AID are distinguished in the enzyme structure [37] (Figure 3):

- Cytidine deaminase domain (residues 20–110) [38];
- Nuclear localization signal (NLS) in the N-terminal region (residues 1–30) [39];
- Nuclear export signal (NES) in the C-terminal region (residues 183–198) [39];
- Interactions sites for
  ○ CTNNBL1 (residues 39–42) [40];
  ○ RNF126 (residues 88–116) [41];
  ○ histone chaperone Spt6 (residues 6–10) [42].

**Figure 4:**
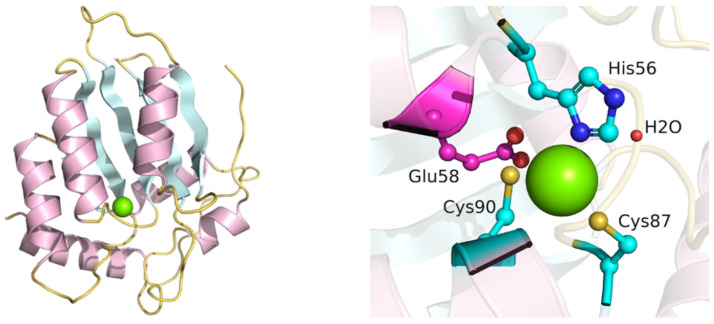
Three-dimensional structure of AID (PDB ID: 5W0U). The Zn atom (green sphere) is coordinated by His56, Cys87 and Cys90 (cyan). Glu58 (magenta) serves as the proton shuttle. Note: Ala58 in the crystal structure was computationally replaced with Glu58 using PyMOL v3.0.

The tertiary AID structure is represented by a five-stranded β-sheet surrounded by six α-helices (α1–β1–β2–α2–β3–α3–β4–α4–β5–α5–α6), forming the backbone of the molecule [37]. The active site contains a Zn-binding domain with the consensus sequence H[AV]E-x[24–36]-PCxxC, where x is any amino acid residue [43]. The residues His56, Cys87 and Cys90 bind the Zn atom, while residue Glu58 plays the role of a proton shuttle (Figure 4). The functional significance of these residues is described in [38,44,45]. It was shown that the double mutant form H56R/E58Q did not possess deamination activity in vitro [44]. Mutant forms H56Y and C87R could not support CSR and SHM processes, as was shown with spleen B-cells and fibroblasts, respectively [38]. Mutant forms H56R and E58K also did not have deamination activity, while retaining the ability to bind single-stranded DNA [45].

**Figure 5:**
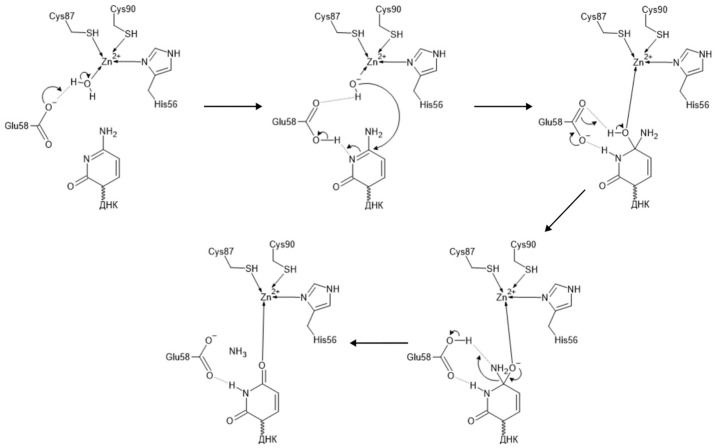
The cytidine deaminase-catalyzed mechanism of cytidine deamination; amino acid residue numbers correspond to AID.

The cytidine deaminase-catalyzed mechanism of cytidine deamination is a highly conserved process that was first determined for *E. coli* cytidine deaminase (CDA) [46]. Cytidine deaminases deaminate cytidine residue to uridine by catalyzing the exchange of amino group to oxygen. The deamination is conducted by a glutamate residue (Glu58 in AID) in the active site, as well as a coordinated Zn atom and water molecule forming zinc hydroxide (ZnOH)^+^ (Figure 5). When cytidine is correctly positioned in the active site, the oxygen atom of zinc hydroxide performs a nucleophilic attack at the C6 atom of cytidine, while the N3 atom of cytidine forms a hydrogen bond with the carboxyl group of catalytic glutamate residue. Further events lead to the protonation of N3 and the exocyclic amino group NH_2_ with formation of a good leaving group NH_3_^+^. Ammonia’s elimination leads to the formation of uridine residue and a non-coordinated Zn^2+^ ion. A new water molecule enters the active site, leading to the protonation of the carboxyl group of catalytic glutamate and the formation of the zinc hydroxide (ZnOH)^+^ necessary for the next stage of catalysis.

## 3. Investigation of Functionally Significant AID Amino Acid Residues

The effect of several amino acid residue substitutions in AID’s processivity (defined as the number of cytosine residues deaminated per substrate-binding event) was studied by Pham P. and co-authors [47]. Several groups of amino acid substitutions were studied: active site substitutions (N51A and Y114A), substrate specificity loop 7 substitutions (F115A, C116A, E117A, R119A, K120A, E122A) and substitutions in helix α6 (R171A, Q175A, R178A) that potentially interact with the substrate and loop 3 substitutions, namely the residue Lys52 that is putatively located on the 3′-side of the deoxycytidine (K52A). Substitutions N51A and Y114A significantly decreased AID’s processivity and deamination activity. Later, it was shown that residue Tyr114 interacts with the oxygen atom of the 5′-phosphate group of the deaminated cytidine residue, while Asn51 forms a hydrogen bond with the 3′–hydroxyl group, facilitating the correct positioning of cytidine in the enzyme active site [48]. Amino acid substitutions in loop 7 (residues 115–122) had little effect on processivity. Substitutions in helix α6 and K52A in loop 3 led to an approximately 2-fold lower processivity [47].

The functional significance of amino acid residues situated beyond the catalytic center, NLS or NES domains was investigated in murine AID [49]. The following residues were chosen for substitution to alanine: Asp45, Phe46, Gly47, His48 (Tyr48 in human AID), Leu49, Arg50, Asn51, Lys52, Ser53 (Asn53 in human AID), Gly54, Cys55. It was shown that the substitution N51A, as well as double and triple substitutions D45A/N51A, R50A/N51A and D45A/R50A/N51A, led to a complete loss of AID’s deamination activity, while substitutions G47A, H48A, L49A and R50A led to a significant reduction. Mutant forms K52A, G54A, C55A and D45A/R50A exhibited reduced deamination activity, whereas D45A, S53A and D45A/F46A had a deamination activity comparable to that of the wild-type enzyme. Comparison of the deamination activity of these mutant forms with their ability to induce CSR and SHM showed that their activity in these processes did not correlate with DNA deamination activity. The mutant forms H48A, L49A, R50A and N51A, despite a low DNA deamination activity, showed a CSR activity similar to that of the wild-type enzyme. The N51A mutant form exhibited conserved CSR activity while lacking DNA deamination activity. It should be noted that almost all of the studied mutant forms, except S53A, had a significantly decreased SHM activity [49]. It was suggested that a decrease in DNA deamination activity, alongside conserved CSR activity, for mutant forms containing substitutions between residues G48 and N51 might be related to disruption of single-stranded DNA binding, which is probably not necessary for CSR. This hypothesis is supported by data showing the altered substrate specificity of mutant forms S38A and S43P, which exhibited high levels of deamination activity in two non–hotspot DNA motifs, GGC and CGC, where the wild-type enzyme is relatively inactive [50]. For human AID, it was also shown that amino acid substitution N51A caused a significant decrease in (but not a complete loss of) DNA deamination activity and the enzyme’s ability to initiate CSR [51].

**Figure 6:**
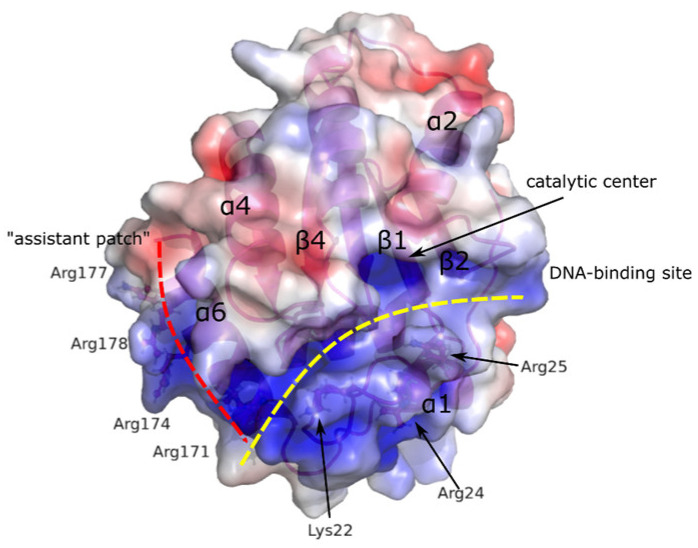
Surface charge distribution of AID and relative positions of the catalytic center, positively charged DNA-binding site and “assistant patch” (adapted from [48]).

Human AID was shown to preferentially recognize and deaminate structured substrates in vitro, in particular, substrates containing G-quadruplexes or branched DNA structures with single-stranded overhangs that mimic switch regions of mammalian immunoglobulin genes [48]. AID crystal structure analysis allowed one to suggest the existence of two DNA-binding sites in the enzyme structure. The first DNA-binding site (referred to as a substrate channel) is responsible for the binding of single-stranded C-containing DNA and is formed by loops α1–β1, β2–α2 and β4–α4, with loop β4–α4 previously identified as a recognition loop important for enzyme specificity [51,52]. The additional positively charged surface on the α6 helix (Arg residues in the 170–178 region) was termed the “assistant patch”. Thus, authors distinguished a bifurcated substrate-binding surface separated by a negatively charged loop β4–α4 in AID’s structure (Figure 6). The presence of this bifurcated substrate-binding surface might explain the recognition of structured substrates through the simultaneous binding of two adjacent overhangs [48]. It was also shown that the binding to G-quadruplex-containing substrates leads to AID’s cooperative oligomerization. For AID mutant forms containing amino acid substitutions in the substrate-binding channel (triple substitutions K22E/R24S/R25E and double substitutions R50E/R52E), a complete loss of deamination activity was observed on DNA substrates with one or two single-stranded DNA overhangs. Mutations R171E, R174E, R174S, R171D/R174E, R177D/R178E in the “assistant patch” region led to a significant reduction in AID deamination activity on DNA substrates with two single-stranded DNA overhangs, while the activity on substrates with one overhang remained unchanged, indicating the assistant role of this region in recognizing structured substrates with multiple overhangs [48]. Experiments on AID-deficient splenic B-cells showed the absence of CSR activity for these mutant forms.

It was demonstrated that AID interacts with a number of cytoplasmic proteins, including histone chaperone Spt6. The inhibition of Spt6 expression in B-cells led to a significant decrease in AID CSR activity while enhancing its SHM activity. Amino acid residues 6–10 in the N-terminal region of AID participate in the interaction with the C-terminal region of Spt6. The substitution M6A disrupted the interaction between AID and Spt6 and also caused the loss of AID’s ability to promote CSR and SHM. In contrast, other substitutions in the N-terminal region of AID (G23S, V18S/R19V, W20K and R24W) did not affect its binding to Spt6. Substitutions N7A, R9A, K10A also led to a decrease in rgw CSR and SHM activity of AID, while R8A, conversely, increased it [42].

The amino acid residue Lys22 is presumed to participate in the regulation of AID activity. Its substitution to Arg led to a loss of AID’s CSR activity while preserving its SHM and deamination activity; AID transport to the nucleus was also unaffected. Substitutions of other Lys residues did not affect AID’s ability to promote CSR [53].

AID forms a complex with the 32 kDa subunit of replication protein A (RPA2), which specifically binds the WRCY (W = A/T, R = A/G, Y = C/T) motif in single-stranded DNA during transcription. For this interaction AID must be phosphorylated [54]. Additionally, AID forms a complex with cAMP-dependent protein kinase A (PKA) in B-cells’ cytoplasm [55]. Murine AID isolated from B-cells is phosphorylated on Ser38 and Tyr184, with Ser38 located within a PKA consensus phosphorylation site, RRX (S/T) (where X is any amino acid). Another potential phosphorylation site exists at residue Thr27 in the AID sequence [56]. Similar phosphorylation sites are present in human AID [55]: a small fraction (5–15%) of human AID expressed in B-cells is phosphorylated on Ser38, and the majority of phosphorylated AID is associated with chromatin [57]. AID is also phosphorylated by a family of protein kinases C (PKC) on Ser38 and Thr140 in vitro [58] and on highly conserved residue Ser3 in vitro and in B-cells [59]. Residue Thr140 is not a phosphorylation site of PKA.

The substitution S3A did not affect AID’s catalytic activity. Mutant forms S3A and S3D possessed increased CSR activity when expressed in B-cells compared to AID WT. Additionally, the S3A mutant form showed increased SHM activity when expressed in fibroblasts. The regulation of AID phosphorylation on Ser3 is mediated by protein phosphatase 2A (PP2A), and its inhibition leads to a decrease in CSR activity of AID WT and, to a lesser extent, the S3A mutant form [59].

The deamination activity of murine AID towards single-stranded DNA is conserved after the substitution of residues Ser38, Thr27 and Tyr184 to Ala. However, mutant forms T27A and S38A are not phosphorylated by PKA in vitro, cannot interact with RPA, lack deamination activity towards double-stranded DNA and fail to support CSR when expressed in AID^−/−^ B-cells, unlike the Y184A mutant form [56]. In agreement with these data, mutant forms of human AID, T27A and S38A, possess a significantly reduced ability to undergo phosphorylation both in vitro and in vivo, while the double mutation T27A/S38A causes a complete loss of this ability [55].

Amino acid substitutions S38A and T140A, double substitution S38A/T140A [58] and substitution S38D [57] did not lead to a loss of deamination activity in human AID. Mutant forms S38A and T140A undergo phosphorylation on residues Thr140 and Ser38, respectively, indicating that these residues are phosphorylated independently of each other. The S38A substitution [58] compromised AID’s ability to induce both SHM and CSR, while the T140A substitution primarily affected SHM [58]. The S38D mutant form also had a decreased ability to induce CSR compared to AID WT [57].

It has also been determined that AID is phosphorylated on amino acid residues Ser41 and Ser43, with only one of the residues Thr27, Ser38, Ser41 or Ser43 phosphorylated in each AID molecule. The substitution S43P is known to be linked to human HIGM2 [60]. Mutant forms S43A, S38D, S43D possess a deamination activity towards ssDNA comparable to that of AID WT, while S38A, S41A, S41D and S43P exhibit decreased activity. According to the authors, these data suggest that AID’s ability to deaminate ssDNA did not depend on phosphorylation or the presence of negatively charged amino acid residues in phosphorylation sites. The listed mutant forms retained their ability to deaminate dsDNA, even in the absence of RPA. Mutant forms S38A and S43P showed altered mutation spectra compared to AID WT [50].

AID interacts with a nuclear protein CTNNBL1 (also known as NAP), which, in turn, interacts with the CDC5L component of the Prp19 complex of the pre-mRNA spliceosome. Substitutions A39G/T40G/S41Q/F42V considerably decreased AID’s ability to interact with CTNNBL1. These substitutions did not disrupt AID’s subcellular localization, deamination activity or oligomerization, but they compromised AID’s ability to conduct IgV gene diversification when expressed in AID^−/−^ cells and to induce CSR in vivo. These data suggest that interaction with CTNNBL1 facilitated the specific binding of AID to DNA in immunoglobulin loci. Additionally, AID’s phosphorylation on Ser38 was not required for its interaction with CTNNBL1, and vice versa, the aforementioned substitutions did not interfere with Ser38 phosphorylation or AID’s interaction with RPA [40].

## 4. Investigation of Natural AID Polymorphic Variants

SNPs are widespread in the human population, and the relationship between SNPs and the etiology of some human diseases, as well as understanding their role in pthe redisposition to oncological and neurodegenerative disorders, remains an open area of research. SNP-associated amino acid substitutions can lead to either a loss of functional properties or the disruption of protein–protein interactions. The NCBI dbSNP database (https://www.ncbi.nlm.nih.gov/snp/) (accessed on 18 September 2024) contains information about more than 4500 SNPs in the human AID gene. For some of these SNPs, their properties and catalytic activity have been researched experimentally.

Hyper-IgM syndrome type 2 (HIGM2) is characterized by a deficiency in the CSR and SHM of IgV genes, as well as abnormal germinal centers [22]. Different types of amino acid substitutions associated with HIGM2 development have been identified. Some affect AID’s interaction with its substrate, while others affect AID’s three-dimensional structure. The first group comprises substitutions of amino acid residues that lie close to the protein surface, R24W, S83P, S85N, A111E, R112C/R112H, L113P and R174S; the second group includes substitutions M6T, F11L, F15L, S43P, W80R, L98R, L106P, I136K, M139T and F151S [47]. Among these, mutant forms S43P, L98R, R174S, F15L and R112C partially retain catalytic activity, with S43P being the most active. Additionally, mutant forms L98R, R174S and F15L possess mutation spectra similar to those of wild-type AID. It seems like the AID mutant forms’ activity does not depend on their ability to bind DNA substrate: mutant forms R174S and R112C bound the substrate cooperatively, as did AID WT, but with a lower affinity, while R112H and L113P (which are catalytically inactive) bound the substrate with an affinity comparable to that of AID WT. S43P has a 4–fold higher affinity to the substrate than AID WT; inactive mutant forms W80R and L106P bind DNA cooperatively, while L98R binds non-cooperatively [45].

In the cell, AID shuttles between the cytoplasm and the nucleus. Its transfer into the nucleus is enabled by a weak nuclear localization signal (NLS) located in the N-terminus, while its export into the cytoplasm is facilitated by a nuclear export signal in the C-terminus and depends on Exportin 1 (CRM1) [39]. Mutations found in the AID gene of patients with HIGM2 that caused a reading frame shift or premature stop codon (such as R190*, where * is a stop codon) in the C-terminus of AID result in an enzyme that retains its ability to initiate SHM while losing CSR-activity in vitro [38,61] and in vivo [62]. Moreover, substitutions of conserved residues Leu and Phe in the NES (L189A, F193A, L196A и L198A) disrupt the nuclear export of AID and significantly decrease its ability to conduct CSR, at the same time increasing its SHM activity. Meanwhile, other substitutions in the C-terminus of AID (L172A, R190A, D191A, A192G, R194A, T195A и G197A) do not have any significant effect on its activity. It was shown that the substitution L196A, as well as truncation of the C-terminal region of AID, does not affect AID’s ability to introduce mutations into DNA [63].

The residue Arg24 is located in the NLS [39] and may also interact with the sugar-phosphate backbone of the DNA substrate [45]. The mutation R24W disrupts AID’s transport into the nucleus and leads to a loss of AID’s ability to initiate both CSR and SHM [38], while its deamination activity is conserved [39]. The substitution M6T also causes a loss of deamination [45] and CSR and SHM activity [64]. Mutant forms of AID containing substitutions S3G and K10R partially retained their SHM and CSR activity [38]. It is worth noting that mutant forms Y13H, V18R, V18R/R19V, W20K и G23S of murine AID retained the ability to conduct CSR but lost SHM activity and the ability to enter the nucleus (except G23S, which accumulates in the nucleus) [65].

Amino acid residues Phe11 and Phe15, despite being located in the NLS, lie inside of the protein globule and may participate in maintaining the structural integrity of AID. The mutant form F11L does not have deamination activity. The substitution of Phe15 to Leu leads to a partial retention of AID’s deamination activity and its specificity towards the WRC motif, as well as the ability to cooperatively bind single-stranded DNA [45]; additionally, this substitution does not affect the subcellular localization of murine AID [66].

The substitution M139V is linked to changes in AID’s three-dimensional structure and causes both the loss of its ability to initiate SHM and CSR [38] and the disruption of its transfer into the nucleus [39]. The M139V mutant form also does not bind Spt6 [42]. Table 1 summarizes known information on AID mutant forms and polymorphic variants.

## 5. In Silico Evaluation of SNPs Effect on AID Functions

The NCBI dbSNP database (https://www.ncbi.nlm.nih.gov/snp/) (accessed on 18 September 2024) contains information about more than 4500 SNPs in the human AID gene. Currently, there are a large number of bioinformatical approaches available to predict the effect of single-nucleotide substitutions on protein functions. Using multiple tools for the prediction of the SNPs’ consequences helps increase the accuracy of the analysis and evaluate the hypothetical ability of SNPs to affect protein functions.

Among the 4500 SNPs deposited in the NCBI dbSNP database, 208 cause amino acid substitutions in the AID primary sequence. These substitutions have been analyzed to estimate their effect on AID’s properties using the dbNSFP 4.7a database [23,24] and several applications: SIFT4G [25], PolyPhen-2 HDIV and PolyPhen-2 HVAR [26], PROVEAN [27], MetaSVM/MetaLR [28], M-CAP [29], REVEL [30], CADD [31] and DANN [32]. Additionally, an evaluation of amino acid residues conservation in the AID structure was performed using the ConSurf Server [33] based on multiple alignment (using the Clustal Omega algorithm [34]) of AID sequences from different species obtained from the UniProt database. For several algorithms (SIFT4G, PolyPhen-2 HDIV and PolyPhen-2 HVAR, PROVEAN, MetaSVM/MetaLR and M-CAP), the effect of SNPs was assigned automatically based on the calculated prediction scores and presented in the query output. For other algorithms (REVEL, CADD and DANN), only the calculated score was presented. The SIFT4G score ranges from 0 to 1. An amino acid substitution was predicted to be damaging if the score was ≤0.05 and tolerated if the score was >0.05. The PolyPhen-2 score ranges from 0.0 (tolerated) to 1.0 (deleterious). Variants with scores ranging from 0.0 to 0.15 were predicted to be benign, those with scores from 0.15 to 1.0 were predicted to be possibly damaging, and variants with scores from 0.85 to 1.0 were more confidently predicted to be damaging (termed “probably damaging” in Table 2 of this review). The PROVEAN score has a predefined threshold of −2.5. If the predicted score for an SNP was equal to or below this threshold, the variant was predicted to have a “deleterious” effect. If the score was above the threshold, the variant was predicted to have a “neutral” effect [27]. For the MetaSVM algorithm, the score threshold is 0.0, and for MetaLR, it is 0.5. SNPs with a predicted score below this threshold are considered to be tolerated, while those with a score above the threshold are considered to be damaging [28]. The M-CAP scores range between 0 and 1. SNPs with a predicted score below the threshold of 0.025 were considered benign, while those above the threshold were considered damaging. For the REVEL, CADD and DANN algorithms, threshold values from [67] were used. For REVEL, SNPs with a score below 0.29 were considered benign, while those above 0.644 were considered pathogenic. For CADD, SNPs with a score below 22.7 were considered benign, while those above 25.3 were considered pathogenic.

Table 2 showcases information for SNPs that led to amino acid substitutions with a negative effect on the enzyme properties based on data from at least six of the nine used applications; SNPs that have been studied experimentally (including M6T, K10R and F15L) were also added.

Analysis of the data makes it possible to conclude that among the 208 SNPs that led to amino acid substitutions in the AID primary structure, 62 (30%) may potentially have a significant negative impact on the functioning of AID. Of these 62 substitutions with a negative effect, 17 have been described in the literature. There is evidence that substitutions M6T, F15L, R24W, S43P, W80R, S83P, S85N, C87R, C87S, L106P, A111E, R112C, L113P, I136K, M139T and F151S are associated with HIGM2’s development [47].

It should be noted that for the described mutant forms M6T, K10R and F15L, a low (K10R) or moderate (M6T, F15L) negative effect was predicted. These residues are located in the NLS region. The mutant form K10R has been shown to partially retain SHM and CSR activities [38]. For F15L, conserved deamination activity was demonstrated [45], while the M6T substitution led to a loss of deamination [45] and CSR and SHM activity [64]. Therefore, it may be necessary to consider not only substitutions with a high predicted negative effect but also those with a moderate effect (predicted by 4 to 5 applications). In this case, the number of substitutions that may negatively affect AID’s properties would increase to 106, constituting 51% of all substitutions.

## 6. Conclusions

AID plays a major role in the processes of antibody diversification, SHM and CSR. The expression of the enzyme variants with increased or reduced activity may lead to the risk of the development of various pathologies. At present the number of studies investigating the role of AID’s polymorphic variants in the development of pathology is low. The study of AID/APOBEC enzymes, particularly their polymorphic variants, holds significant promise for advancing our understanding of immune regulation, disease mechanisms and therapeutic interventions. The following key perspectives and future research directions can be highlighted.

*Mechanistic insights into AID’s dysregulation*

- Structural–functional relationships—Further high-resolution structural studies of AID mutants could elucidate how specific SNPs alter enzyme–substrate interactions, oligomerization, and interactions with regulatory partners like Spt6 or RPA.
- Post-translational modifications (PTMs)—The systematic investigation of PTMs (e.g., phosphorylation at Ser3/Ser38) and their combinatorial effects on AID’s activity could reveal fine-tuned regulatory mechanisms.

*Disease associations and biomarker development*

- HIGM2 and beyond—Expanding clinical studies to correlate uncharacterized SNPs (e.g., those with a “moderate” predicted impact) with HIGM2’s severity or atypical presentations.
- Cancer predisposition—Exploring whether AID hypermutation variants (e.g., R174S, F15L) contribute to early oncogenic events in B-cell malignancies or other cancers.

*Computational and Experimental Integration*

- AI-driven predictions—Leveraging machine learning to prioritize high-risk SNPs by integrating multi-omics data (e.g., epigenetics, protein interactomes) with existing tools.
- Functional screens—Using CRISPR-based mutagenesis in B-cell models to validate predicted pathogenic variants and uncover novel functional domains.

*Therapeutic Opportunities*

- Precision targeting—Developing small-molecule inhibitors or stabilizers to modulate AID’s activity in pathologies (e.g., inhibitors for AID-driven lymphomas, activators for HIGM2 patients with hypomorphic variants).

*Evolutionary and Translational Perspectives*

- Cross–species comparisons—Investigating AID polymorphisms in non-human primates to identify conserved regulatory motifs or compensatory mechanisms.
- Vaccine design—Harnessing AID’s role in SHM to engineer B-cells for broader antibody responses in vaccines or infectious disease models.

Taken together, the dual role of AID—as a guardian of immune diversity and a potential driver of disease—underscores the need for a multidisciplinary approach. Bridging computational predictions with mechanistic studies and clinical data will not only refine the risk stratification for immune disorders but also open opportunities for targeted therapies. Future research should prioritize the translational validation of SNPs’ effects and exploit AID’s unique biology for therapeutic innovation.

## Figures and Tables

**Table 1:** Effect of amino acid substitutions (artificial and SNP variants) on the functioning of AID.

Residue	Location	Effect	Ref.
Investigation of functionally significant amino acid residues (artificial mutant forms)			
M6A N7A R8A R9A K10A	Spt6 interaction site	disrupted interaction with Spt6; reduced CSR and SHM activity (excluding R8A)	[42]
Y13H V18R V18R/R19V W20K G23S	NLS	conservation of CSR activity and loss of SHM activity and ability to enter the nucleus (excluding G23S)	[65]
S3A S3D	phosphorylation sites	increased CSR and SHM activity	[59]
T27A S38A S38D S41A S41D S43A S43D Y184A T27A/S38A S38A/T140A	phosphorylation sites	conservation of deamination activity towards ssDNA for T27A, S38A, S38D, S43A, S43D and Y184A, loss of CSR-activity for T27A, S38A and S38D, reduced ability to undergo phosphorylation, disrupted interaction with RPA for T27A and S38A; according to other sources, decrease of deamination activity for S38A, S41A and S41D	[55,56,57,58]
A39G/T40G/S41Q/F42V	CTNNBL1 interaction site	disrupted interaction with CTNNBL1, compromised ability to potentiate IgV gene diversification with conserved deamination activity	[40]
H56R/E58Q H56Y H56R E58K C87R	catalytic center	loss of deamination activity; loss of CSR and SHM activity for H56Y and C87R; loss of deamination activity with conserved ability to bind ssDNA for H56R and E58K	[38,44,45]
N51A Y114A	active site	decreased deamination activity; decreased CSR and SHM activity for N51A	[47,49]
F115A C116A E117A R119A K120A E122A	loop 7 (β4–α4)	no effect on deamination activity	[47]
R171A Q175A R178A	helix α6	decreased deamination activity	[47]
K52A	loop 3 (β2–α2)	decreased deamination activity	[47,49]
D45A/N51A R50A/N51A D45A/R50A/N51A	loop β2–α2	complete loss of deamination activity; decreased CSR and SHM activity	[49]
G47A H48A L49A R50A	loop β2–α2	decreased deamination activity; conserved CSR activity for H48A, L49A and R50A and decreased CSR activity for G47A; decreased SHM activity	[49]
G54A C55A D45A/R50A	loop β2–α2	decreased deamination activity; conserved CSR activity for G54A and C55A and decreased CSR activity for D45A/R50A; decreased SHM activity	[49]
D45A S53A D45A/F46A	loop β2–α2	no effect on deamination activity; decreased CSR and SHM activity for D45A and D45A/F46A, conserved CSR and SHM activity for S53A	[49]
K22E/R24S/R25E R50E/R52E	DNA-binding site, loops α1–β1, β2–α2 и β4–α4	complete loss of deamination activity on DNA substrates with one or two single-stranded overhangs; lack of CSR activity	[48]
R171E R174E R174S R171D/R174E R177D/R178E	“assistant patch”, helix α6	significantly reduced deamination activity on DNA substrates with two single-stranded overhangs; conserved activity on substrates with one single-stranded overhang; lack of CSR activity	[48]
L189A F193A L196A L198A	NES	disrupted nuclear export of AID; significantly reduced CSR activity with increased SHM activity	[63]
L172A R190A D191A A192G R194A T195A G197A	C-terminus	no effect on AID activity	[63]
**Investigation of natural AID polymorphic variants**			
R24W	NLS	disrupted transport of AID into the nucleus, loss of CSR and SHM activity	[38,39]
M6T	NLS	loss of deamination, CSR and SHM activity	[45,64]
S3G K10R	NLS	partially conserved CSR and SHM activity	[38]
F11L	NLS	loss of deamination activity	[45]
F15L	NLS	partially conserved deamination activity and ability to bind ssDNA, no effect on subcellular localization	[45,66]
S83P S85N A111E R112C R112H L113P R174S	protein surface	partially conserved deamination activity for R174S and R112C with decreased affinity for the substrate; loss of deamination activity for S83P, S85N, A111E, R112H and L113P; unaffected affinity for the substrate for R112H and L113P	[47]
S43P W80R L98R L106P I136K M139T F151S		partially conserved deamination activity for S43P and L98R; loss of deamination activity for W80R, L106P, I136K, M139T and F151S; increased affinity for the substrate for S43P	[45]
M139V	loop 9 (β5–α5) inside the protein globule	putative change in AID’s three-dimensional structure, loss of CSR and SHM activity, disrupted transport into the nucleus and binding to Spt6	[38,39,42]
R190 *	NES	conserved SHM activity and loss of CSR activity	[38,61,62]

* is a stop codon.

**Table 2:** Predicted effect of amino acid substitutions in the AID sequence caused by SNPs.

SNP	ConSurf Conservation Score ^1^	Buried/ Exposed ^2^	Functional/ Structural ^3^	SIFT4G	PolyPhen-2 HDIV	PolyPhen-2 HVAR	PROVEAN	MetaSVM	MetaLR	M-CAP	REVEL	CADD	Ref.
M6T	6	E		D	B	B	D	T	T	D			[45,64]
K10R	6	E		T	B	B	N	T	T	D	B	B	[38,42]
F15L	9	B	S	D	P	B	D	T	T	D	P		[45,66]
K16Q	4	E		T	D	P	N	D	D	D		P	
R24W	7	E		T	D	D	D	D	D	D	P	P	[22,38,39,42,45,64,68]
39R24Q	7	E		D	D	D	N	T	D	D		P	
E26A	5	E		T	D	D	D	T	T	D	P	P	
Y28H	7	B		D	D	D	D	D	D	D	P	P	
Y31C	7	B		D	D	P	D	D	D	D	P	P	
R50C	4	E		T	D	D	D	T	T	D		P	
R50G	4	E		T	D	D	D	D	D	D		P	
E58K	9	E	F	D	D	D	D	T	T	D	P	P	[45]
L60P	5	B		T	D	P	D	T	T	D	P	P	
R77H	4	E		D	D	D	D	T	T	D		P	
T79P	9	B	S	T	D	D	D	D	D	D	P	P	
T79S	9	B	S	D	D	D	D	D	D	D	P	P	
T79I	9	B	S	D	D	D	D	T	T	D	P	P	
W80R	7	B		D	D	D	D	D	D	D	P	P	[22,38,45,64,68,69]
S83P	9	B	S	D	D	D	D	D	D	D	P	P	[45,64]
W84G	9	B	S	D	D	D	D	D	D	D	P	P	
W84R	9	B	S	D	D	D	D	D	T	D	P	P	
S85R	9	E	F	D	D	D	D	D	D	D	P		
S85N	9	E	F	D	D	D	D	D	D	D	P	P	[45,64]
P86L	9	E	F	D	D	D	D	D	D	D	P	P	
C87S	9	B	S	D	D	D	D	D	D	D	P	P	[45,64]
C87R	9	B	S	D	D	D	D	D	D	D	P	P	[38,45,64,68]
A91P	9	B	S	D	P	P	D	D	D	D	P	P	
A91V	9	B	S	D	D	P	D	T	T	D	P	P	
A91T	9	B	S	D	P	P	D	D	T	D		P	
F97V	8	B		D	D	D	D	D	T	D	P	P	
N103S	8	E	F	D	D	D	D	T	T	D		P	
L106P	9	B	S	D	D	D	D	D	D	D	P	P	[22,45,64,68,69]
F109S	6	B		D	D	D	D	T	T	D	P	P	
A111E	8	B		D	D	P	D	T	T	D	P	P	[45,64]
A111V	8	B		D	D	P	D	T	T	D	P	P	
R112C	9	E	F	D	D	D	D	D	D	D	P	P	[38,45,69,70]
L113P	9	B	S	D	D	D	D	D	D	D	P	P	[45,64]
Y114D	8	B		D	D	D	D	D	D	D	P	P	
G125R	8	B		D	D	D	D	D	D	D	P	P	
G125E	8	B		D	D	D	D	D	D	D	P	P	
L126P	9	B	S	D	D	D	D	D	D	D	P	P	
R127W	7	E		D	D	D	D	T	T	D			
L129P	9	B	S	D	B	B	D	D	D	D	P		
L129V	9	B	S	D	P	P	D	D	D	D			
G133R	8	E	F	D	D	D	D	D	D	D		P	
V134M	7	B		D	P	P	N	D	T	D		P	
I136K	6	B		D	P	P	D	T	T	D	P		[45]
M139T	9	B	S	D	D	D	D	D	D	D	P	P	[45,64,69,71]
M139V	9	B	S	D	D	D	D	D	D	D	P		[22,38,39,42,45,64,68]
M139I	9	B	S	D	D	D	D	D	D	D	P	P	
D143V	7	E		D	D	D	D	T	T	D	P	P	
D143Y	7	E		D	D	D	D	D	D	No data	P	P	
Y144H	7	B		D	D	P	D	T	T	D		P	
Y144C	7	B		D	P	P	D	T	T	D		P	
Y146C	5	E		D	D	D	D	T	T	D	P	P	
Y146F	5	E		D	D	D	D	T	T	D		P	
C147F	8	B		D	D	D	D	D	D	D	P	P	
C147Y	8	B		D	D	D	D	D	D	D	P	P	
W148C	9	B	S	D	D	D	D	D	D	D	P	P	
T150A	7	E		D	P	P	D	T	T	D		P	
F151S	9	B	S	D	D	D	D	D	D	D	P	P	[22,45,64,68,69]
V152L	9	E	F	D	D	D	N	D	D	D	P	P	

^1^ Higher scores correspond to conserved residues (on a scale from 1 to 9, where 9 is conserved, 1—variable) [33]. ^2^ Buried (B)—residues located inside the protein globule, exposed (E)—residues located on the protein surface (based on the neural network prediction of residue contact numbers [33]). ^3^ Functional (F)—highly conserved and exposed residue, structural (S)—highly conserved and buried residue [33]. SIFT4G—Score ranges 0 ÷ 1, D—damaging (≤0.05), T—tolerated (>0.05); PolyPhen-2 HDIV, PolyPhen-2 HVAR—Score ranges 0.0 ÷ 1.0, D—probably damaging (0.85 ÷ 1.0), P—possibly damaging (0.15 ÷ 1.0), B—benign (0.0 ÷ 0.15); PROVEAN—Score threshold −2.5, D—deleterious (≤−2.5), N—neutral (>−2.5); MetaSVM4—Score threshold 0.0, MetaLR4—Score threshold −0.5, D—damaging (<threshold), T—tolerated (>threshold); M-CAP Score ranges 0 ÷ 1, B—benign (<0.025), D—damaging (>0.025); REVEL—Threshold values used from [67], P—pathogenic (>0.644), B—benign (<0.29), cells highlighted with yellow contain values outside of these ranges; CADD—Threshold values used from [67], P—pathogenic (>25.3), B—benign (<22.7), cells highlighted with yellow contain values outside of these ranges. The background color reflects the predicted level of the amino acid substitutions impact: from red for damaging to green for tolerated/benign.

## Data Availability

Data are available from E.A.K. (E-mail: ekat.koveshnikova@gmail.com) and A.A.K. (Tel.: +7-(383)-363-5174, e-mail: Sandra-k@niboch.nsc.ru) upon request.
